# The seasonality, steroid use, and lower ratio of neutrophil to lymphocyte associated with bacteremia of *Listeria monocytogenes* in Japan from 2010 to 2019: a case–control study

**DOI:** 10.1186/s12879-021-06926-7

**Published:** 2021-12-05

**Authors:** Yusuke Watanabe, Itaru Nakamura, Yuri Miura, Hidehiro Watanabe

**Affiliations:** 1 Department of Infection Prevention and Control, Tokyo Medical University Hospital, 6-7-1 Nishishinjuku, Shinjuku-ku, Tokyo, 160-0023 Japan; 2 Department of Microbiology Laboratory, Tokyo Medical University Hospital, Tokyo, Japan

**Keywords:** *Listeria monocytogenes*, Listeriosis, Seasonality, Steroid, Ratio of neutrophil to lymphocyte, Adult, Asia

## Abstract

**Background:**

Despite having a high mortality rate, Asian studies about the characteristics of adult listeriosis are limited. We investigated the incidence of listeriosis per admissions, associated factors, and rate of mortality in listeriosis, compared with non-listeriosis.

**Methods:**

We recorded the incidence of listeriosis per 10,000 admissions and conducted a case–control study from January 1, 2010, to December 31, 2019, at Tokyo Medical University Hospital (TMUH) in Japan. Cases were defined as adult with listeriosis that was bacteremia due to *L. monocytogenes*. Controls, defined as adult with non-listeriosis bacteremia due to other pathogens, were matched by age and clinical department to cases. We analyzed differences in seasonality, including warm season (defined as the period from May to October), medication including steroids, laboratory findings, and mortality. The odds ratio and p value between the cases group and control group were calculated using a chi-square test and Fisher’s exact test.

**Results:**

The incidence of listeriosis per 10,000 admissions to TMUH was 0.51. Eleven patients, excluding one neonate, were included in the case group. Twenty-six patients, excluding one patient because of contamination and one patient because of insufficient medical record, were included in the control group. Listeriosis onset was associated with the warm season (90.9% vs. 53.8%; p = 0.033), steroid use (54.5% vs. 19.2%; p = 0.042), and a lower ratio of neutrophils to lymphocytes (9.46 vs. 18.44; p = 0.015). The 30-day mortality rate of listeriosis was similar to non-listeriosis (18.3% vs. 19.2%; p = 0.619).

**Conclusion:**

The incidence of listeriosis per admissions in this study was similar to that in other Asian countries. Factors associated with listeriosis were the warm season, steroid use, and a lower ratio of neutrophils to lymphocytes. Additionally, the 30-day mortality rate was similarly high in both the listeriosis and non-listeriosis groups.

## Background

*Listeria monocytogenes* is a ubiquitous Gram-positive rod in the environment. The invasive infection caused by *L. monocytogenes,* which can lead to bacteremia and meningitis, is called listeriosis. Although listeriosis has generally been recognized as a foodborne infection that develops outside the hospital, foodborne listeriosis acquired in a hospital has also been reported [1–3]. Several outbreaks attributable to a variety of foods have been reported in Italy, the United Kingdom, Denmark, and the United States over the past few decades [4–8]. Although the incidence per 100,000 people as a foodborne illness is lower than that of other pathogens like *Campylobacter* sp., *Salmonella* sp., and *Shigella* sp., rates of hospitalization and death due to listeriosis are high in the United States [9–12]. In Europe, listeriosis with high case-fatality is one of the most serious foodborne diseases [13]. Comparably, two studies of listeriosis from China also suggested that high mortality rates exist among adults [14, 15]. However, adequately researched studies of listeriosis in Asian populations are more limited than those from Western countries, due in part to underreporting [16]. We therefore endeavored to investigate the incidence of listeriosis per admissions, associated factors, and mortality in listeriosis, compared with non-listeriosis in Japanese hospitalized patients.

## Methods

Tokyo Medical University Hospital (TMUH) is a 1015-bed university tertiary hospital located in Tokyo, Japan. We conducted a case–control study of adults with listeriosis, defined as bacteremia of *L. monocytogenes,* or non-listeriosis, defined as bacteremia due to other pathogens, infection from January 1, 2010, to December 31, 2019, at TMUH. The study population included all hospitalized patients. Cases were defined as patients with listeriosis. Controls were patients with non-listeriosis and were selected according to age and clinical department of treatment to match each case of listeriosis. Exclusion criteria were patients under the age of 18 years, contamination confirmed by infectious disease doctors in TMUH based on the presence of coagulase-negative *Staphylococci*, *Bacillus* sp., or *Corynebacterium* sp. in blood culture bottles without clinically sufficient evidence, refusal of participation in the study, and insufficient medical records (e.g., those with no records of underlying disease, treatment and/or outcome). We defined community-associated listeriosis as listeriosis occurring before hospital admission. In the case of listeriosis occurring after admission, the number of days of hospitalization from admission to bacteremia was noted. We examined the incidence of listeriosis per 10,000 admissions for observation. Differences in the clinical characteristics were analyzed; these characteristics comprised of sex, aged ≥ 70 years, seasonality, underlying conditions [including diabetes, chronic kidney disease (CKD), cirrhosis, solid tumor, autoimmune diseases, lung disease, active gastrointestinal disease, coronary artery disease, hypertension, arrhythmia, intravascular device, chronic heart failure, and pregnancy], symptoms of infection, community-associated bacteremia or the number of days of hospitalization from admission to bacteremia, use of immunosuppressant including steroids, methotrexate (MTX) and adalimumab use, acid inhibitor use, oral iron, blood count including the ratio of neutrophils to lymphocytes, laboratory findings, antibiotics use, and mortality.

Blood cultures were performed using the BACTEC FX system (Becton, Dickinson, and Co., Franklin Lakes, NJ, USA), and strains were identified with the MicroScan WalkAway 96 (Beckman Coulter Inc., Brea, CA, USA) and microflex LT/SH (MALDI Biotyper; Bruker Corporation, Billerica, MA, USA) systems. The entry of causative microorganisms was identified by infectious disease doctors. The warm season was defined as the period from May to October, and the cold season was defined as the period from November to April after collecting the dates of onset month.

Statistical analysis was performed using the Statistical Package for the Social Sciences for Windows, version 26 (IBM Corporation, Armonk, NY, USA). The test of normality was performed using the Shapiro–Wilk test. Group comparisons were performed using two-sample *t*-tests and Welch’s test for normally distributed continuous variables and the Mann–Whitney *U* test for non-normally distributed continuous variables. Differences in proportions between the case group and the control group (e.g., the odds ratio and the p value) were calculated and compared using a chi-square test and Fisher’s exact test. A significance threshold of 0.05 was adopted for all statistical analyses.

This study was approved by the TMUH institutional review board (approval no. T2020-0379); after obtaining this approval, we disclosed this study to patients via the TMUH homepage and provided the option to refuse participation in this study.

## Results

There were a total of 236,689 admissions over 10 years to TMUH. Listeriosis was confirmed in 12 patients, including 11 adults. The incidence of listeriosis was 0.51 per 10,000 admissions to TMUH.

One neonate was excluded from the case group since this study focused only on adults. Therefore, 11 adults with listeriosis were selected for inclusion in the case group. Two patients were excluded from the control group after initially matching to the case group in age and clinical department of treatment; one patient was identified as a contamination case and the other patient had insufficient information in their medical history. Thus, 26 patients were identified for inclusion in the control group.

The clinical characteristics of the case group are shown in Table 1. Underlying diseases in the listeriosis group included chronic kidney disease (CKD) (n = 3), autoimmune disease (n = 3), solid tumor (n = 3), hematologic malignancy (n = 3), intravascular device (n = 3), diabetes (n = 2), hepatitis C (n = 2), and cirrhosis (n = 1). In the non-listeriosis group, 15 patients had hypertension, 12 had coronary artery disease, 7 had diabetes, and 7 had CKD. In the listeriosis group, 4 patients were treated with only steroids, 2 patients were treated with both steroids and methotrexate, and 1 patient was treated with adalimumab. Although 5 patients were taking steroids in the non-listeriosis group, none were treated with MTX or a biological drug such as adalimumab. Two cases presented with complicated central nervous system infections, including one with meningitis and one with a brain abscess, in the listeriosis group. One patient in the non-listeriosis group developed psoas abscess as a complication of bacteremia. The involved clinical departments consisted of the departments of neurosurgery, neurology, cardiology, nephrology, respiratory medicine, urology, and rheumatology.

**Table 1:** Characteristics of 11 cases of listeriosis in adult

Sex	Age (years)	Days from admission to bacteremia	Month	Underlying disease	Immunosupressant or biological therapy	Symptoms	N/L	Complication	Antibiotics	Outcome
M	71	8	July	Cirrhosis due to hepatitis C, brain tumor, diabetes	Bethamethasone	Fever, consciousness disturbance	11,025/831	None	None	Death
F	72	CA	October	Rheumatoid arthritis, sjogren syndrome, hepatitis C	Prednisolone, Methotrexate	Fever, headache, joint pain	< 400/ < 400	None	ABPC/SBT → ABPC	Lived
M	64	CA	March	Febrile neutropenia, prostate cancer	Hydrocortisone	Fever, diarrhea	6868/754	None	CFPM → ABPC → AMPC	Lived
M	87	CA	June	Diabetes, giant cell arteritis	Prednisolone	Fever, dizziness	9334/535	Meningitis	CTRX + VCM → ABPC	Death
M	77	CA	June	CKD due to nephrotic syndrome, amyloidosis, total gastric resection	Prednisolone	Fever	14,789/753	None	CTM + CLDM → ABPC + SMX/TMP	Lived
M	82	27	May	Rectal ulcer, SSS on pacemaker	None	Fever, bloody stool	7039/848	None	LVFX → ABPC	Lived
M	88	CA	July	CKD, aortic stent graft, valve replacement, atrophic gastritis,	None	Fever, diarrhea	3901/795	None	CTRX → ABPC	Lived
M	63	CA	June	CKD on hemodialysis, renal cancer, synthetic graft	None	Fever, vomiting, diarrhea	4537/715	None	MEPM + VCM → ABPC	Lived
F	36	CA	June	Pregnancy, ulcerous colitis	Adalimumab	Fever, bloody stool	12,039/2293	None	ABPC/SBT → ABPC → MEPM	Lived
M	79	61	September	Relapsing polychondritis	Prednisolone, Methotrexate	Fever, abdominal pain, diarrhea, convulsion	6064/1880	Brain abscess	PIPC/TAZ → ABPC + GM	Lived
M	84	CA	October	Combined pulmonary fibrosis and emphysema	None	Fever, dyspnea	5364/474	None	CTRX → ABPC	Lived

*M* male; *F* Female; *CA* community-associated; *CKD* chronic kidney disease; *N* neutrophil count; *L* lymphocyte count; *ABPC/SBT* ampicillin/sulbactam; *ABPC* ampicillin, *CFPM* cefepime; *AMPC* amoxicillin; *CTRX* ceftriaxone; *VCM* vancomycin; *CTM* cefotiam; *CLDM* clindamycin; *SMX/TMP* sulfamethoxazole/trimethoprim; *LVFX* levofloxacin; *MEPM* meropenem; *PIPC/TAZ* piperacillin/tazobactam; *GM* gentamicin

**Fig. 1:**
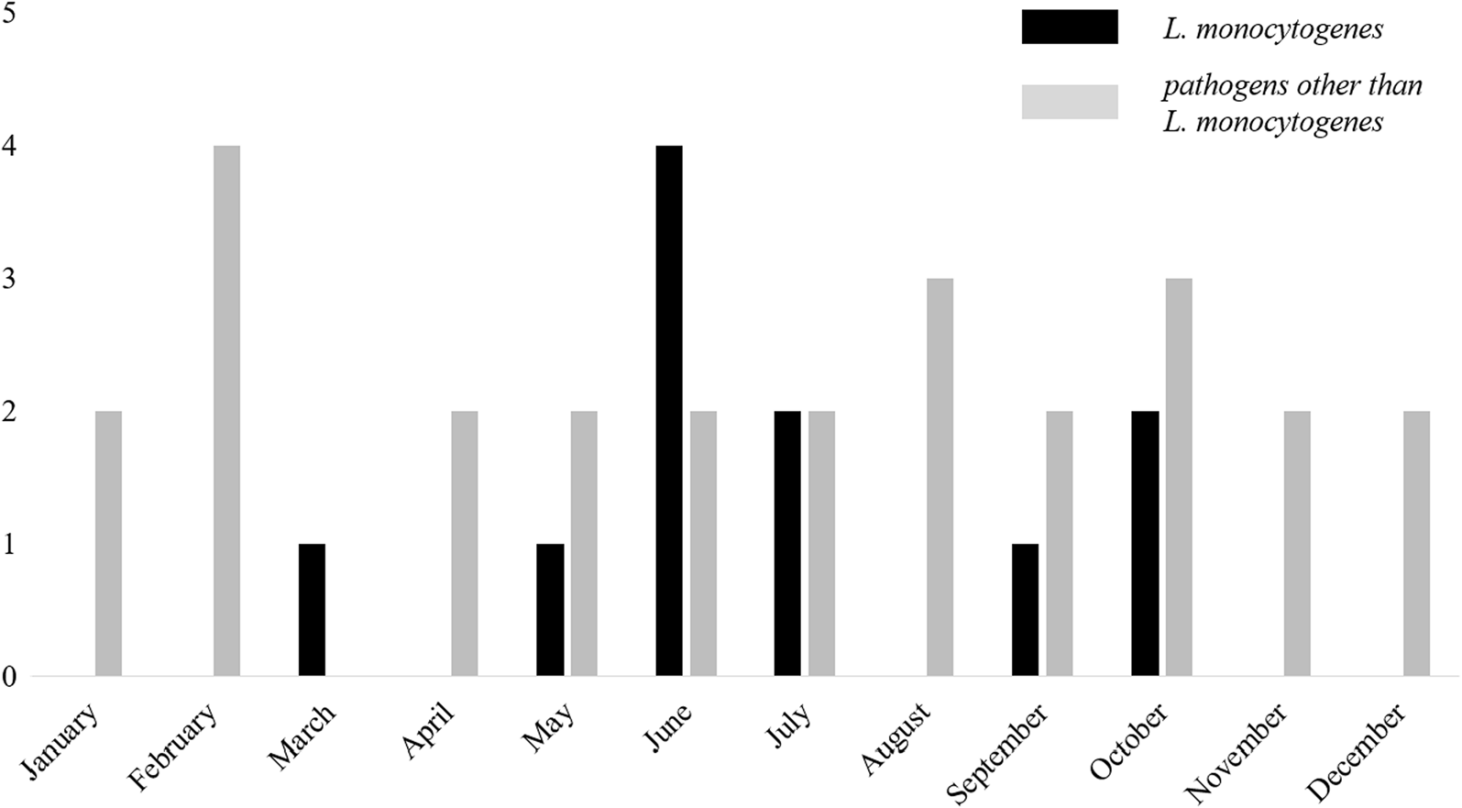
Distribution of 11 admission months of *L. monocytogenes* bacteremia and 26 admission months of the pathogens other than *L. monocytogenes* bacteremia

The distribution of onset month among the 11 listeriosis cases is shown in Fig. 1 and, notably, 10 cases of listeriosis had developed during the warm season. Cephalosporins, to which *L. monocytogenes* is inherently resistant, were administered in five cases as initial empiric therapy; one case without initial empiric therapy progressed to meningitis and 2 of the 5 cases died. The 30-day mortality rate in the listeriosis group was 18.2%.

The causative microorganisms in the control group were *Staphylococcus aureus* (29.6%), *Escherichia coli* (18.5%), *Klebsiella pneumoniae* (11.1%), *Enterococcus faecalis* (11.1%), coagulase-negative *Staphylococci* (11.1%), extended-spectrum β-lactamase-producing *Enterobacteriaceae* (11.1%), *Pseudomonas aeruginosa* (3.7%), *Enterobacter aerogenes* (3.7%), *Serratia marcescens* (3.7%), and group B streptococci (3.7%).

The case group and control group were compared to calculate the odds ratios and p-value of clinical characteristics (Table 2). The onset of the listeriosis group was statistically significantly more frequent in the warm season than in the cold season when compared with the non-listeriosis group (90.9% vs. 53.8%; p = 0.033). Underlying diseases did not differ between the groups, but the listeriosis group was taking more steroids (54.5% vs. 19.2%; p = 0.042). Although the mean counts of neutrophils and lymphocytes were not different between the groups, the ratio of neutrophils to lymphocytes was lower in the listeriosis group than in the non-listeriosis group (9.46 vs. 18.44; p = 0.015). The entries of bacteremia were less clear in the listeriosis group than the non-listeriosis group (100.0% vs. 34.6%; p < 0.001). The 30-day mortality rate of the listeriosis group was similar to that of the non-listeriosis group (18.3% vs. 19.2%; p = 0.619).

**Table 2:** Factors and outcomes associated with listeriosis and non-listeriosis

Characteristics	Cases (n = 11)	Controls (n = 26)	Odds Ratio (95% confidence interval)	P value
Male	7 (63.6)	16 (61.5)	0.914 (0.212–3.939)	0.603
Female	4 (36.4)	10 (38.5)		
Age, ≥ 70 years	8 (72.7)	25 (96.1)	0.107 (0.01–1.175)	0.070
Warm season	10 (90.9)	14 (53.8)	8.571 (0.954–77.008)	0.033
Underlying disease, n (%)				
Diabetes	2 (18.3)	7 (26.9)	0.603 (0.104–3.507)	0.454
CKD	3 (27.3)	7 (26.9)	1.018 (0.209–4.965)	0.639
Cirrhosis	1 (9.1)	0 (0.0)		0.297
Solid tumor	3 (27.3)	3 (11.5)	2.875 (0.479–17.239)	0.236
Hematogical malignancy	0 (0.0)	1 (3.8)	0.694 (0.559–0.862)	0.703
Organ transplant	0 (0.0)	0 (0.0)		
Autoimmune disease	3 (27.3)	3 (11.5)	2.875 (0.479–17.239)	0.236
HIV	0 (0.0)	0 (0.0)		
Lung disease	1 (9.1)	5 (19.2)	0.420 (0.043–4.087)	0.41
Stroke	0 (0.0)	5 (19.2)	0.656 (0.511–0.843)	0.151
Active gastrointestinal disease	4 (36.4)	3 (11.5)	4.381 (0.785–24.453)	0.099
Coronary artery disease	4 (36.4)	12 (46.2)	0.667 (0.156–2.843)	0.429
Hypertension	4 (36.4)	15 (57.7)	0.419 (0.098–1.794)	0.235
Arrhythmia	4 (36.4)	5 (19.2)	2.4 (0.500–11.519)	0.241
Intravascular device	3 (27.3)	4 (15.4)	2.063 (0.376–11.309)	0.339
Chronic heart failure	2 (18.3)	7 (26.9)	0.603 (0.104–3.507)	0.454
Perinatal	1 (9.1)	0 (0.0)		0.297
Medicine, n (%)				
Steroid	6 (54.5)	5 (19.2)	5.040 (1.085–23.419)	0.042
MTX	2 (18.3)	0 (0.0)		0.083
Adalimumab	1 (9.1)	0 (0.0)		0.297
Acid inhibitor	10 (90.9)	20 (76.9)	3.0 (0.317–28.434)	0.31
Oral iron	1 (9.1)	3 (11.5)	0.767 (0.071–8.299)	0.659
Symptoms, n (%)				
Fever	11 (100.0)	21 (80.8)	1.524 (1.186–1.958)	0.151
Diarrhea	4 (36.4)	2 (7.7)	6.857 (1.031–45.604)	0.051
Bloody stool	2 (18.3)	2 (7.7)	2.667 (0.325–21.872)	0.341
Central nervous system manifestation	2 (18.3)	0 (0.0)		0.083
Blood test, mean (Min–Max, SD)				
Neutrophil, /μL	7396 (399*-14,789, 4108)	10,225 (2892–23,016, 5298)		0.146
Lymphocyte, /μL	1024 (399*-2293, 595)	709 (99–1519, 394)		0.264
Neutrophil/Lymphocyte	9.46 (1–19, 5.662)	18.44 (5–56, 13.032)		0.015
Hb, g/dL	11.2 (8.5–14.2, 1.6)	11.6 (6.0–15.6, 2.4)		0.921
Platelet, × 10^3^/μL	177.5 (32.0–429, 127.0)	169.8 (56.0–332.0, 85.6)		0.821
AST, U/L	32.0 (14–51, 14.526)	29.48 (16–49, 10.638)		0.618
ALT, U/L	19.09 (4–40, 11.22)	22.10 (7–58, 13.870)		0.584
LDH, U/L	326.45 (220–620, 119.277)	305.9 (159–764, 145.781)		0.347
CK, U/L	150.67 (19–514, 155.482)	70.56 (21–209, 52.415)		0.152
Na, mmol/L	135.82 (128–145, 5.231)	138.77 (131–150, 4.364)		0.096
CRP, mg/dL	8.735 (0.6–17.9, 5.64)	12.008 (0.3–31.5, 9.5385)		0.418
BS, mg/dL	154.9 (75–420, 116)	132.84 (65–193, 38.322)		0.353
Entry of causative microorganisms				
CRBSI	0 (0.0)	6 (23.1)		0.099
UTI	0 (0.0)	6 (23.1)		0.099
IE	0 (0.0)	2 (7.7)		0.488
Cholecystitis	0 (0.0)	1 (3.8)		0.703
Iliopsoas muscle abscess	0 (0.0)	1 (3.8)		0.703
Pacemaker infection	0 (0.0)	1 (3.8)		0.703
Unknown	11 (100.0)	9 (34.6)		< 0.001
Admission to ICU	2 (18.3)	5 (19.2)	0.933 (0.152–5.739)	0.661
No administration of effective for empiric therapy	6 (54.5)	10 (38.5)		0.16
30 day mortality, n (%)	2 (18.3)	5 (19.2)	0.844 (0.137–5.220)	0.619

*SD* standard deviation; *CKD* chronic kidney disease; *HIV* human immunodeficiency virus; *MTX* methotrexate; *Hb* hemoglobin; *AST* aspartate aminotransferase; *ALT* alanine aminotransferase; *LDH* lactase dehydrogenase; *CK* creatinine kinase; *Na* sodium; *CRP* C-reactive protein, *BS* blood sugar; *CRBSI* catheter related blood stream infection; *UTI* urinary tract infection; *IE* infective endocarditis; *ICU* intensive care unit

## Discussion

To our knowledge, this study is the first case–control study to compare cases of listeriosis and non-listeriosis. In Asia, especially in Japan, studies of characteristics of listeriosis are limited. A nationwide survey conducted between 1980 and 2002 in Japan reported that the incidence of *L. monocytogenes* infection was 0.65 cases per one million people [17], although the incidence per 10,000 admissions is unclear because listeriosis is not designated as an infectious disease of concern in Japan. Only meningitis of *L. monocytogenes* is under sentinel surveillance in Japan. However, bacteremia of *L. monocytogenes* is not under either sentinel surveillance or notifiable disease surveillance. A study from Taiwan in 2015 reported that the incidence of listeriosis is 1.25 per 10,000 admissions [18], while one from Korea in 2018 reported that the incidence of listeriosis was 0.31 per 10,000 admissions [19]. Our incidence of listeriosis is similar to these prior Asian reports.

Angelo et al. reported that the median incubation period of invasive listeriosis was 11 days and the maximum incubation period was 30 days in pregnancy-associated cases [20]. Goulet et al. reported that the median incubation period of invasive listeriosis was 8 days and the maximum incubation period was 67 days in pregnancy-associated cases [21]. In this study, we found that 3 cases of listeriosis occurred after hospital admission. The number of days for each case from admission to bacteremia was 8 days, 27 days, and 61 days. Because the third case with prednisolone and methotrexate was a non-pregnancy case and was diagnosed after an unusually long period of 61 days after admission, there was a possibility of hospital-associated listeriosis. The study of the seasonality of listeriosis is lacking and results are variable. One study from Taiwan reported that more than 36.7% of patients acquired invasive listeriosis in the spring [18], while research from England and Wales reported that summer was the predominant season of listeriosis onset [22]. In Israel, the incidence in non-perinatal women was 64.2% in the hot season (May–October) [23]. A study from France documented an increase in cases during the summer [24]. Our study also contended that listeriosis occurs more frequently in the warm season. The reason for increased numbers of listeriosis cases in the warm or hot season is unclear, although Vasilev et al. hypothesized that there is a link with the concomitant increase in gastrointestinal infection [23]. Although *L. monocytogenes* infections were more prevalent during the warmer season in a cold-smoked fish processing plant in Japan [25], the seasonality of foodborne listeriosis is unclear. More epidemiological investigation of listeriosis is needed.

The MONALISA study reported that the mean counts of total leucocytes and blood polymorphonuclear cells were respectively 10,920/μL and 8400/μL in patients with bacteremia caused by *L. monocytogenes* [9]. However, no study of the ratio of neutrophils to lymphocytes in patients with listeriosis has been performed. Our cases of listeriosis showed a significant difference in this ratio and in the use of steroids relative to patients in the control group. Although steroid use increases neutrophil counts because neutrophil is released from the pool of mature neutrophils in the bone marrow [26], the ratio of neutrophils to lymphocytes in our listeriosis group was lower than that in the non-listeriosis group. This finding may assist in diagnosing listeriosis early.

A systematic review from researchers in China reported patients with non-perinatal listeriosis had a mortality rate of about 23.8% [27]. The 30-day mortality rate of their listeriosis group was similar to that of the non-listeriosis group in our study (18.3% vs. 19.2%). Moreover, one non-fatal case in our study developed a brain abscess, suggesting that clinicians should carefully monitor listeriosis in patients with certain risk factors.

There are some limitations to this study. First, this study included only 11 cases of listeriosis and this limited report, arising from a single center, may have been unable to detect significant risk factors of listeriosis. Because the data on listeriosis in Japan are limited, large-scale and multicenter studies should be conducted. Second, we could not comprehensively assess the presence or absence of meningitis in our study population because the analysis of cerebrospinal fluid was performed in only one case. However, the proportions of *L. monocytogenes* that were isolated from blood and cerebrospinal fluid in an outbreak report were 84% in blood and 5% cerebrospinal fluid; thus, there are likely few cases of undiagnosed meningitis in our study [7]. Third, the exclusion of the only neonatal patient from the case group means the results of this study cannot be extended to neonatal or pediatric patients. A future study focusing on the neonatal period is required. Fourth, the serotype or virulence factors of *L. monocytogenes*, such as internalin A and B, with the ability to invade human epithelial colorectal adenocarcinoma cells were not checked [28]. Further investigation into gene transcription and invasiveness among *L. monocytogenes* is needed.

## Conclusions

The incidence of listeriosis in this study was similar to that in other Asian countries. Onset in the warm season, steroid use, and a lower ratio of neutrophils to lymphocytes were risk factors for listeriosis compared with non-listeriosis. Additionally, because the 30-day mortality rate was similarly high in both the listeriosis and non-listeriosis groups, clinicians need to take prompt action to identify the offending organism and establish a treatment plan.

## Data Availability

The datasets supporting the conclusions of the current study are available at the Tokyo Medical University Hospital. The data that support the findings of this study are available on request to the correspondence author. (Itaru Nakamura, Email: task300@tokyo-med.ac.jp).
